# Excess Glucose Impedes the Proliferation of Skeletal Muscle Satellite Cells Under Adherent Culture Conditions

**DOI:** 10.3389/fcell.2021.640399

**Published:** 2021-03-01

**Authors:** Yasuro Furuichi, Yuki Kawabata, Miho Aoki, Yoshitaka Mita, Nobuharu L. Fujii, Yasuko Manabe

**Affiliations:** Department of Health Promotion Sciences, Graduate School of Human Health Sciences, Tokyo Metropolitan University, Tokyo, Japan

**Keywords:** satellite cell, proliferation, self-renewal, glucose, primary culture

## Abstract

Glucose is a major energy source consumed by proliferating mammalian cells. Therefore, in general, proliferating cells have the preference of high glucose contents in extracellular environment. Here, we showed that high glucose concentrations impede the proliferation of satellite cells, which are muscle-specific stem cells, under adherent culture conditions. We found that the proliferation activity of satellite cells was higher in glucose-free DMEM growth medium (low-glucose medium with a glucose concentration of 2 mM) than in standard glucose DMEM (high-glucose medium with a glucose concentration of 19 mM). Satellite cells cultured in the high-glucose medium showed a decreased population of reserve cells, identified by staining for Pax7 expression, suggesting that glucose concentration affects cell fate determination. In conclusion, glucose is a factor that decides the cell fate of skeletal muscle-specific stem cells. Due to this unique feature of satellite cells, hyperglycemia may negatively affect the regenerative capability of skeletal muscle myofibers and thus facilitate sarcopenia.

## Introduction

Skeletal muscle has the ability to regenerate after injury. The main players of muscle regeneration are muscle-specific stem cells, known as satellite cells, which reside between the sarcolemma and basal lamina of myofibers. Satellite cells normally stay quiescent and have a low metabolic rate but initiate the myogenic program in response to muscle injury (Brack and Rando, 2012). Activated satellite cells express myoblast determination protein 1 (MyoD), a key transcription factor in myogenesis, and proliferate as myoblasts before differentiating and fusing to repair damaged muscles (Tajbakhsh, 2009). A portion of satellite cells does not commit to muscle differentiation but remains as stem cells by asymmetric division to maintain the satellite cell pool; this is termed as self-renewal (Collins et al., 2005). The ability of satellite cells to regenerate skeletal muscle is crucial not only for repairing muscle injury but also for maintaining muscle mass (Keefe et al., 2015). Since satellite cells are applicable to regenerative medicine for muscle diseases (Sacco et al., 2008), it is necessary to investigate the mechanism underlying satellite cell behavior.

Glucose is an essential energy substrate and an anabolic precursor for various mammalian cells. In anaerobic glycolysis, two ATP molecules are generated from one glucose molecule, and the central metabolite pyruvate is used as a substrate in the mitochondrial tricarboxylic acid (TCA) cycle through oxidative phosphorylation. In addition to its catabolic role, glucose is used for the synthesis of nucleotides through the pentose phosphate pathway, which is essential to produce ribose for DNA synthesis during cell division. Proliferating cells, such as cancer cells, prefer glucose as fuel to support their rapid proliferation (Han et al., 2011; Jones and Schulze, 2012; Ito et al., 2017; Luo et al., 2018; Zhou et al., 2018). For these reasons, it is believed that the media of various cultured cells require high concentrations of glucose to improve cell proliferation.

High-glucose media have also widely been used for culturing muscle cells (Shefer and Yablonka-Reuveni, 2005; Pasut et al., 2013). However, here we found that a high-glucose medium was not suitable for culturing satellite cells under adherent conditions. The proliferation activity of satellite cells was higher in a low-glucose medium than in the standard high-glucose medium. Additionally, the population of reserve cells, as indicated by Pax7 expression, was increased by lowering the glucose concentration, suggesting that glucose affects the cell fate determination of satellite cells. High glucose levels disturb important functions of satellite cells, such as cell proliferation and self-renewal. An excessive glucose concentration seems to represent a negative factor for skeletal muscle homeostasis because hyperglycemia is known to induce the impairment of muscle regeneration and atrophy.

## Experimental Procedures

### Animals and Experimental Design

Adult C57BL/6 male mice (8–12 weeks) were used in this study. Mice were fed normal chow and water under standard lighting conditions (12-h:12-h light–dark cycle).

### Myofiber and Satellite-Cell Isolation and Culture

Extensor digitorum longus (EDL) and soleus muscles were isolated and digested using type I collagenase, as described previously (Ono et al., 2012). Collected myofibers were confirmed to be surely clean and not to include other cells such as fibroblasts under the high-magnitude microscope. Single myofibers were incubated with Accutase (Innovative Cell Technologies) for 10 min and cultured on Matrigel-coated dishes. Standard growth medium was composed of high-glucose Dulbecco’s modified Eagle’s medium (DMEM) containing GlutaMAX (Thermo Fisher Scientific), 30% fetal bovine serum (FBS) (BioWest or Nichirei), 1% chicken-embryo extract (CEE) (USBiological), and 1% penicillin–streptomycin. We prepared the low-glucose growth medium using glucose-free DMEM (Thermo Fisher Scientific) supplemented with 1% GlutaMAX to adjust other components with the standard growth medium. Cells were incubated at 37°C under an atmosphere of 5% CO_2_, and media were changed every day, beginning on the third of culture. Myogenic differentiation was induced using the differentiation medium (high-glucose DMEM supplemented with 5% horse serum and 1% penicillin–streptomycin) at 37°C under an atmosphere of 5% CO_2_. For floating culture, isolated myofibers were cultured in the plating medium (DMEM supplemented with 10% horse serum, 0.5% CEE, and 1% penicillin-streptomycin) without incubation of Accutase (Ono et al., 2012).

### Analysis of Cell Proliferation and Differentiation

For the cell proliferation assays, 20 myofibers were cultured in each well (24-well plate) in duplicate, and stained with DAPI or Ki67, a marker of cell proliferation. To detect EdU incorporation, the Click-iT EdU Imaging Kit (Thermo Fisher Scientific) was used according to the manufacturer’s instructions. On day-6 of culture, the cells were treated with 10 μM EdU for 6 h, and fixed in 4%PFA for 15 min.

To analyze the apoptosis of cultured satellite cells, the TUNEL assay was performed using the Click-iT^TM^ Plus TUNEL Assay for in situ Apoptosis Detection, Alexa Fluor^TM^ 594 dye (Thermo Fisher Scientific) according to the manufacturer’s instructions.

For evaluation of myoblast differentiation, cells cultured in low-glucose growth medium for 6-days were passaged in 24-well plates to the same density and cultured for 3-days in differentiation medium. Myosin heavy chain (MHC), a contractile protein expressed in differentiated muscle cells, was stained. The fusion index, the number of nuclei inside myotubes as a percentage of the total number of nuclei, was calculated and compared between glucose concentrations.

### Degradation of Glucose in FBS Using Immobilized Enzymes

Glucose oxidase (GOD) and catalase were dissolved in phosphate-buffered saline (PBS) at 100 and 3,700 U/mL, respectively. The enzyme solution was mixed with Biosurfine-AWP-MRH (Tokyo Gosei Kogyo Co.) at a ratio of 1:10 (v/v) and pasted on a glass slide (Matsunami). Each side of the glass slide was exposed to UV light for 30 min to immobilize the enzymes on the slide. FBS was incubated with the glass slide by gently shaking in a 50-mL tube for 3 weeks at 6°C. The glucose concentration was measured using the glucose (HK) assay kit (Sigma Aldrich), according to the manufacturer’s instructions. Glucose-depleted FBS was filtered through a 0.22 μm polyvinylidene fluoride (PVDF) membrane before use for cell culture.

### Immunostaining

Immunocytochemistry of cultured myoblasts and myotubes was performed as described previously (Ono et al., 2012). Samples were fixed with 4% paraformaldehyde, blocked/permeabilized with PBS containing 0.3% Triton X-100 and 5% goat serum for 30 min at room temperature, and incubated overnight with primary antibodies at 4°C. The following antibodies were used: anti-alpha-actinin (Sigma Aldrich, A7811), anti-Ki67 (Cell Signaling Technology, 9129), anti-MHC (R&D Systems, MAB4470), anti-Pax7 (DSHB, PAX7), anti-MyoD (Santa Cruz Biotechnology, sc-760), and anti-myogenin (Santa Cruz Biotechnology, sc-12732). Immunostained samples were visualized using the appropriate species-specific Alexa Fluor 488- and 594 fluorescence-conjugated secondary antibodies (Thermo Fisher Scientific). The samples were observed and photographed using a Keyence BZ-X800 microscope or a Nikon Ti-U microscope. Images were automatically taken in a scan to cover the entire 24 wells and then tiled. Cell numbers were quantified using the Keyence software BZ-H3C and H3CM.

Immunostaining myofiber-associated satellite cells was performed as described previously (Ono et al., 2012). Briefly, cultured myofibers were fixed with 4% paraformaldehyde, blocked/permeabilized with PBS containing 0.3% Triton X-100 and 10% goat serum for 30 min at room temperature, and incubated overnight with primary antibodies for Ki67 (Cell Signaling Technology, 9129) at 4°C. The primary antibodies were visualized by appropriate species-specific 488 and 594 fluorescence -conjugated secondary antibodies (Thermo Fisher Scientific). Nuclei were stained with VECTASHIELD fluorescent mounting medium containing 4,6-diamidino-2-phenylindole (DAPI; Vector Laboratories, Burlingame, CA, United States).

### Western Blotting

Total protein extracts were obtained from homogenized tissues and cultured cells, and lysed with RIPA or lysis buffer (Manabe et al., 2014). Protein samples were separated by sodium dodecyl sulfate-polyacrylamide gel electrophoresis and transferred to PVDF membranes. The membranes were blocked with Tris–buffered saline containing 5% nonfat dry milk and 0.1 % Tween 20. The membranes were then incubated overnight with the following primary antibodies: anti- β-actin (Cell Signaling Technology, 4967), anti-MHC I (Sigma Aldrich, M8421), anti- MHC II (Sigma Aldrich, M4276), anti-GAPDH (Cell Signaling Technology, 2118), and Phospho-AMPK (Thr172) (Cell Signaling Technology, 2531). Subsequently, the membranes were treated with rabbit (GE Healthcare) or goat (Millipore) secondary antibodies conjugated to horseradish peroxidase. The blots were developed using ECL plus (PerkinElmer Life Sciences) and analyzed with a Luminescent Image Analyzer LAS-4000 mini (GE Healthcare). Data were quantified using the ImageJ software.

### Statistical Analysis

Data are expressed as the mean ± standard error of the mean (SEM). Two-sided unpaired t-tests were used to compare data between the two groups. N in the figure legends indicates the number of mice used in the experiments. The level of significance was set to *p* < 0.05.

## Results

### Glucose Limitation Facilitates the Cell Proliferation of Primary Satellite Cells

**FIGURE 1:**
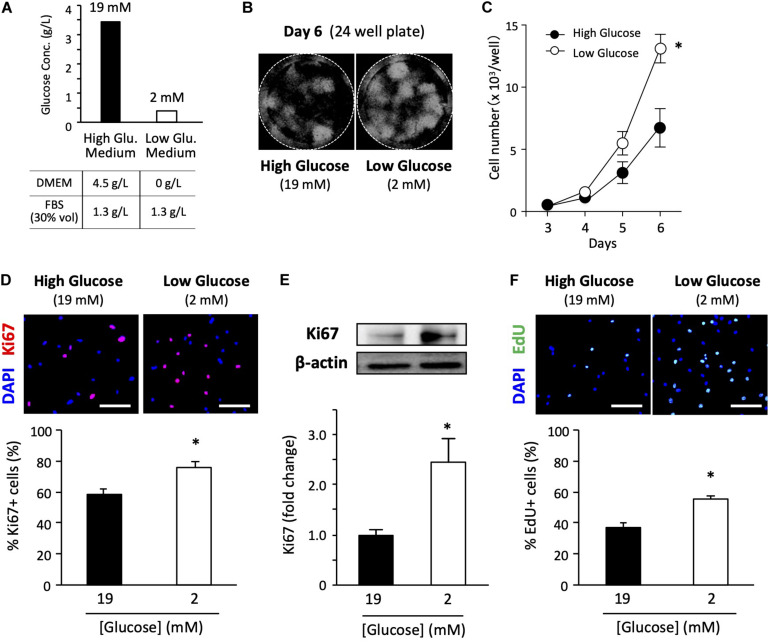
Low-glucose medium increases the proliferation of primary satellite cells. **(A)** Glucose concentration in each growth medium used in this study. **(B)** Proliferation of primary satellite cells in high- and low-glucose media. Satellite cells from 20 myofibers were isolated from EDL and seeded in 24-well plates. Cell nuclei were visualized using DAPI and marked by the Hybrid Cell Count application (Keyence software). All the cells cultured in each well were automatically counted. **(C)** Cell growth curves. Values are presented as the mean ± SEM (*n* = 7). ^∗^*p* < 0.05. **(D)** Immunofluorescence analysis of proliferating cells cultured for 6 days. The population of Ki67-positive cells was quantified in high- and low-glucose media. Scale bars are 100 μm. Values are presented as the mean ± SEM (*n* = 3). ^∗^*p* < 0.05. **(E)** Western blot analysis of Ki67 protein expression in high- and low-glucose media after 6 days of cultivation. Ki67 expression was normalized to that of β-actin. Values are presented as mean ± SEM (*n* = 13). **(F)** Representative images of EdU+ satellite cells and the quantification of the number of EdU+ cells cultured for 6 days in high- and low-glucose media. Scale bars are 100 μm. Values are presented as the mean ± SEM (*n* = 4). ^∗^*p* < 0.05.

In general, high-glucose DMEM is the standard medium for culturing primary satellite cells (Ono et al., 2010, 2012). Therefore, we used a high-glucose medium containing 30% FBS and some other cell culture supplements (Figure 1A). The final glucose concentration was 19 mM in the high-glucose medium containing 30% FBS. We also prepared a growth medium containing a very low glucose concentration using glucose-free DMEM as a basic medium. The low-glucose medium had a final glucose concentration of 2 mM due to carry-over from 30% FBS. Despite the carry-over from FBS, the total glucose concentration in the low-glucose medium was only 10% of that in the high-glucose medium. We concluded that the glucose concentration in the FBS used was about 1.3 g/L (Figure 1A). Detailed information and formulation of the media are cited in Supplementary Table 1. The glucose concentration in serum and CEE were cited in Supplementary Table 2.

To examine the effect of glucose concentration on satellite cell proliferation, we determined cell growth curves in high- and low-glucose media. We cultured satellite cells obtained from 20 myofibers in each 24-well plate for 3, 4, 5, and 6 days before counting cells visualized by DAPI staining (Figure 1B). As shown in Figure 1C, cell proliferation was promoted in the low-glucose medium compared to that in the standard high-glucose medium. A statistically significant difference in cell number was observed on the sixth day of culturing between high and low glucose conditions. Ki67 is a routinely used cell proliferation marker. The ratio of Ki67-positive cells to total cells evaluated by immunohistochemical staining (Figure 1D) and the total expression level of Ki67 protein quantified by immunoblotting (Figure 1E) were significantly elevated for the low-glucose medium compared to the corresponding values for the high-glucose medium after 6 days of culturing. To confirm the change in proliferation due to glucose, the EdU pulse-chase assay was performed under high and low glucose conditions. The satellite cells grown in the low-glucose medium had a higher number of EdU-positive cells compared to that in the high-glucose medium (Figure 1F), suggesting that low glucose facilitates cell proliferation of satellite cells. These data provide direct evidence that glucose restriction facilitates the proliferation of satellite cells.

We also examined whether glucose regulated cell proliferation in a dose-dependent manner. Satellite cells derived from 20 myofibers were cultured for 6 days in media with different glucose concentrations, and the ratio of Ki67-positive cells to total cell number was quantified. Cell proliferation was enhanced in media with a glucose concentration of 8 mM or less (Supplementary Figure 1). To minimize the effects of the adhesive property of the myofibers on the Matrigel and the myoblast migration rate from the myofibers attached to the Matrigel on cell proliferation, we also analyzed the proliferation capacity after passage. The primary cells were first grown from a single dish, and the same number of cells recovered from the dish was re-cultured in high-glucose and low-glucose media. We observed that the cell number and percentage of Ki67-positive cells tended to be higher in the low-glucose medium (Supplementary Figure 2).

Cell number is potentially affected by cell viability and cell death. Therefore, we evaluated the apoptosis of satellite cells in high- and low-glucose media for 6 days using a TUNEL assay. The percentage of apoptotic cells was lower in the low-glucose medium than in the high-glucose medium, indicating that glucose restriction ameliorated cell damage and maintained cell viability (Supplementary Figure 3). We analyzed the cell proliferation of satellite cells while they were still attached to myofibers. Isolated myofibers were cultured in high and low glucose medium for 72 h and then satellite cells were stained for Ki67. The number of proliferating cells, marked as Ki67-positive cells, did not differ between high- and low-glucose conditions in this type of experimental model (Supplementary Figure 4).

We also performed experiments to examine whether low-glucose medium maintained the proliferative activity of the satellite cell-derived myoblasts. When high-glucose medium was used, cell proliferation was inhibited in around 1 week. However, we observed that the cells cultured in the low-glucose medium could be passaged, and they maintained their proliferative activity for more than 2 weeks (Supplementary Figure 5). The passaged cells had the capacity for differentiation, because they fused and expressed MHC protein after being cultured in the differentiation medium (Supplementary Figure 5). We demonstrated that myoblasts cultured in low-glucose medium were able to be frozen, and could proliferate again after thawing. We also observed that cells frozen twice were able to differentiate normally (Supplementary Figure 6).

We examined whether glucose regulates myoblast differentiation. Myoblasts grown in low-glucose medium were passaged and cultured in differentiation medium (5% horse serum in DMEM) at different glucose concentrations for 3 days. The myotubes were fixed, and fusion indices were calculated by staining the MHC. Myoblasts were normally differentiated even when cultured in low-glucose medium, and fusion indexes were similar regardless of glucose concentration (Supplementary Figure 7).

### Glucose Concentration Affects Cell Fate Determination

**FIGURE 2:**
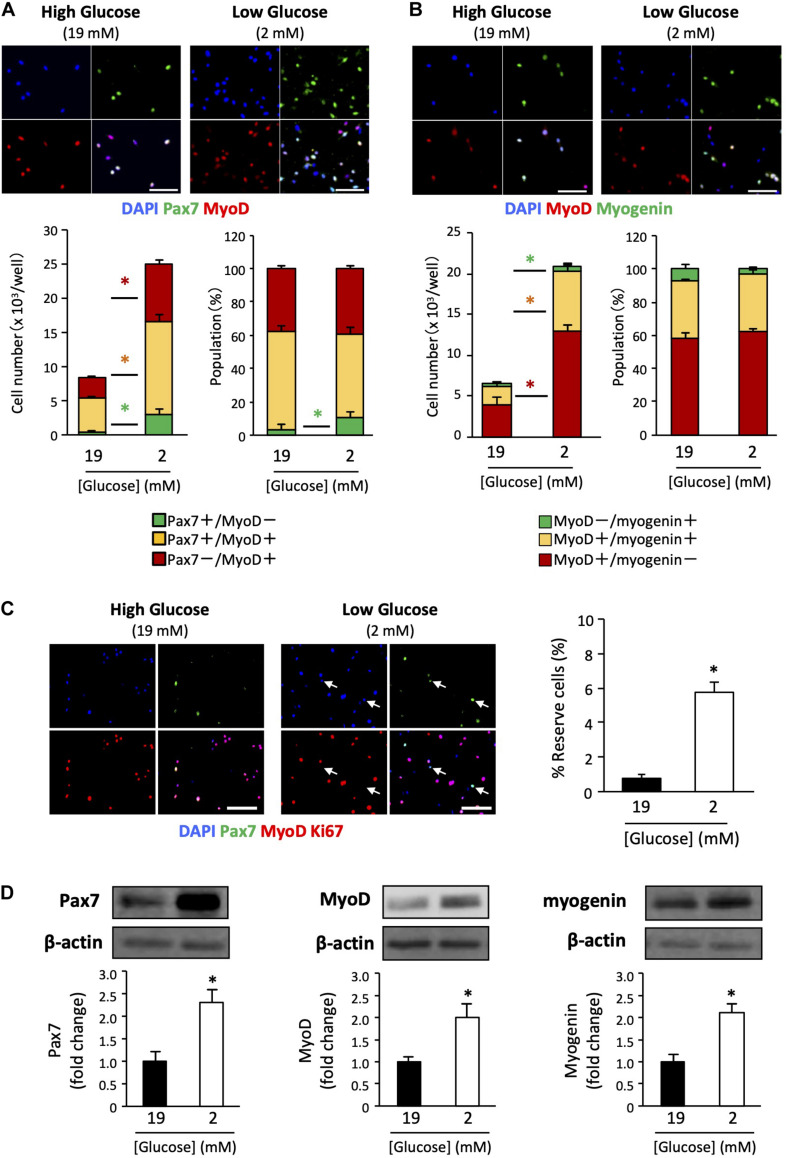
Expression patterns of myogenic factors in satellite cells derived from EDL under low- and high-glucose media. **(A)** Satellite cells stained with Pax7 and MyoD antibodies after 6 days of cultivation. Scale bars are 100 μm. Quantified data are expressed as absolute and relative values. Population of self-renewed cells is indicated by Pax7+/MyoD-. Values are presented as mean ± SEM (*n* = 6). *p < 0.05. **(B)** Satellite cells stained with MyoD and myogenin antibodies after 6 days of cultivation. Scale bars are 100 μm. Values are presented as mean ± SEM (*n* = 6). *p < 0.05. **(C)** Reserve cell frequencies of primary myoblasts cultured in high- and low-glucose growth media. The cells were stained with Pax7, MyoD, and Ki67 antibodies after 6 days of cultivation. Reserve cells, identified as Pax7+/MyoD-/Ki67- cells, were indicated by arrows. Scale bars are 100 μm. The percentages of reserve cells were quantified, and higher in the low-glucose condition than in the high glucose condition. Values are presented as mean ± SEM (*n* = 4). *p < 0.05. **(D)** Western blot analysis of Pax7, MyoD, and myogenin protein expression levels in cells cultured for 6 days in high-and low-glucose media. The expression of all proteins was normalized to that of β-actin. Values are presented as mean ± SEM (*n* = 6). *p < 0.05.

We determined the myogenic status of the satellite cells using myogenic markers. Pax7 is an important transcription factor for maintaining the stemness of satellite cells, and is a recognized self-renewed cell marker (Halevy et al., 2004; Olguin and Olwin, 2004). MyoD is a master regulator of myogenesis that is expressed at the early stage of muscle differentiation (Bentzinger et al., 2012). Myogenin is a differentiation marker expressed during muscle differentiation. Cultured cells were co-immunostained with Pax7 / MyoD or MyoD / myogenin. The Pax7+/MyoD – phenotype reflects self-renewed cells. The Pax7+/MyoD+ phenotype represents cells that are activated/proliferating cells, and the Pax7–/MyoD+ phenotype indicates myogenic committed cells (Brun et al., 2014). Further, the MyoD+ / myogenin – phenotype represents activated/proliferating cells, MyoD+/myogenin+ represents myogenic committed cells, and the MyoD–/myogenin+ phenotype represents cells differentiating cells (very minor population). As shown in Figures 2A,B, the number of cells of each myogenic status was increased in low-glucose media compared to that in high-glucose media, as was the overall cell number. Noteworthily, the higher percentage of Pax7+/MyoD– cells in the low-glucose medium suggests that the self-renewed satellite cells is better maintained under low glucose conditions in vitro (Figure 2A). There were no changes in the populations of activated and committed cells between high and low glucose conditions, as indicated by MyoD and myogenin expression levels (Figure 2B). To examine the effect of glucose on the generation of reserve cells, which are undifferentiated but retain their myogenic potential (Yoshida et al., 1998), the cells were stained for Ki67 in addition to Pax7 and MyoD. The frequency of reserve cells, marked as Pax7+/MyoD-/Ki67- cells was greater in low-glucose medium than in high-glucose medium (Figure 2C). We performed western blotting to quantify the expression of Pax7, MyoD, and myogenin in the cells, showing that these proteins were increasing under the low glucose condition (Figure 2D). The data show that cell proliferation/differentiation was progressing in the experimental situation, while the progression of cell fate was different between cells in 2 vs 19 mM glucose conditions.

### The Low Glucose Condition Inhibited the Expansion of Non-Myogenic Cells and Enabled the Culture of High Purity Muscle Cells

**FIGURE 3:**
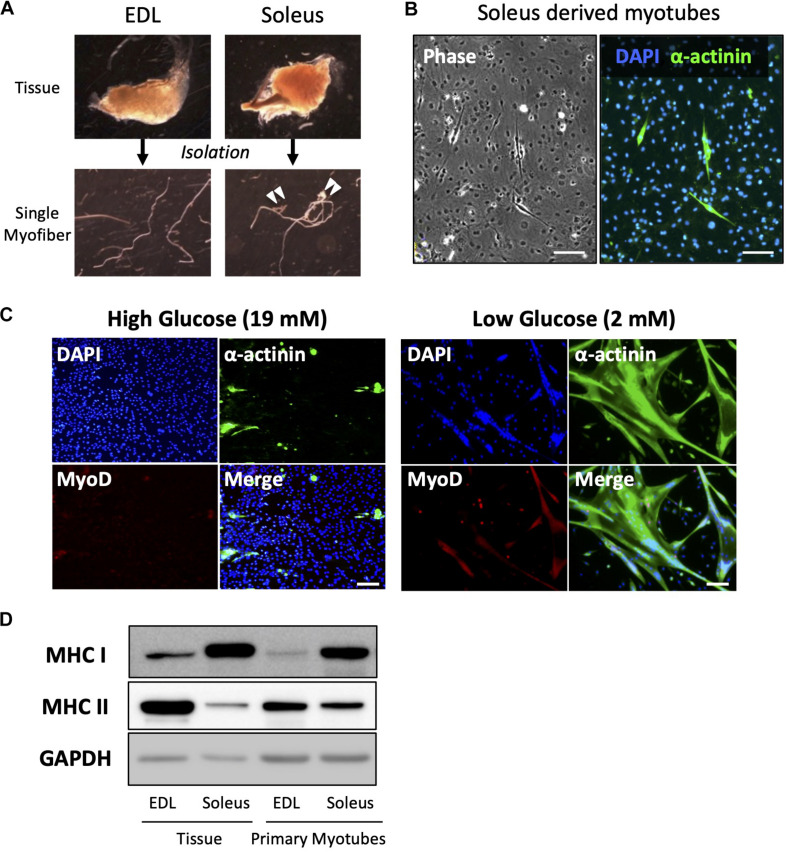
Low-glucose medium enables the culturing of soleus-derived satellite cells in high purity. **(A)** Images of EDL and soleus tissues and single myofibers isolated from each tissue. Soleus muscle contains an Achilles tendon and connective tissues and cells (indicated by white arrowheads). **(B)** Images of phase-contrast (left) and immunofluorescence (right) of α-actinin (green) and DAPI (blue) in cultured satellite cells derived from soleus. Only a few muscle and α-actinin-positive cells were observed, and most of the cells visualized by DAPI were contaminated with non-muscle cells. Scale bars are 100 μm. **(C)** Immunofluorescence analysis of the purity of muscle cells derived from soleus satellite cells. In the standard high-glucose medium, most of the cells were contaminated with non-muscle cells because neither α-actinin nor MyoD (markers of myotubes and myoblasts, respectively) was expressed in the cells. On the contrary, most of the cells in the low-glucose medium expressed α-actinin, suggesting that muscle cells were purified by glucose restriction. Scale bars are 100 μm. **(D)** Western blot analysis of MHC I and II expression in cultured satellite cells isolated from EDL and soleus myofibers. GAPDH was used as house-keeping protein. The cells were differentiated for 5 days before western blotting was performed. The amounts of loaded protein derived from tissues and cell samples were 2.5 μg/lane and 10 μg/lane, respectively.

Primary culture of satellite cells from single myofibers was performed using EDL muscle because the myofibers can be isolated with a low risk of contamination by non-muscle cells or connective tissues (Figure 3A). EDL muscle is mainly composed of type II fibers, which express the MHC II, and shows glycolytic characteristics. Accordingly, myotubes differentiated from satellite cells, which were isolated from EDL fiber, expressed MHC II, which indicates that they are type II myotubes. To obtain slow-type myotubes, we isolated myofibers from soleus, a representative slow-type muscle. It is, however, difficult to isolate clean single myofibers from soleus because most myofibers contain non-muscle cells (Figure 3A); therefore, these myofibers must be eliminated to prevent contamination with non-muscle cells as pointed by previous reports (Rosenblatt et al., 1995; Danoviz and Yablonka-Reuveni, 2012). We cultured satellite cells from soleus myofibers and stained α-actinin, a marker of differentiated muscle cells. Staining revealed that most of the cells were non-muscle cells, such as fibroblasts (α-actinin-negative cells). When satellite cells contaminated with fibroblasts are plated on dishes, the fibroblasts expanded rapidly and inhibited the growth of satellite cells, due to their high capacity for cell proliferation (Figure 3B).

When satellite cells were cultured in a standard high-glucose medium, fibroblasts preferentially grew, as shown above (Figure 3C). Mononuclear cells were observed, but they were not satellite cell-derived myocytes because MyoD, a marker of myogenic cells, was not expressed by them. On the contrary, when satellite cells were cultured in a low-glucose medium, most of them differentiated and expressed α-actinin. This indicated that satellite cells only grew normally in the low-glucose medium in which other cells could not survive (Figure 3C). Using this medium, we successfully cultured soleus-derived satellite cells in high purity and obtained myotubes from both EDL and soleus muscles. As expected, soleus-derived myotubes exhibited slow-type characteristics because they predominantly expressed MHC I instead of MHC II. The expression pattern of the MHC isoforms in primary myotubes was similar to that in muscle tissue (Figure 3D).

### Satellite Cells Survive in Glucose-Depleted Growth Medium

As indicated before, the glucose-free DMEM we used in this study contained a small amount of glucose (2 mM) that originated from the FBS component. To test whether satellite cells can survive under even lower glucose conditions, we degraded the glucose in FBS using the enzymes GOD and catalase. GOD breaks down glucose into gluconic acid and H_2_O_2_ (1). Since H_2_O_2_ is harmful to cells, catalase was used to degrade it to H_2_O and CO_2_ (2).

(1) $$\beta -\text{D}-\text{glucose}+{\text{O}}_{2}+{\text{H}}_{2}\,\text{O}\to \text{D}-\mathrm{gluconic}\,\mathrm{acid}+{\text{H}}_{2}\,{\text{O}}_{2}$$

(2) $$2\,H_{2}\,{\text{O}}_{2}\to 2\,H_{2}\,\text{O}+{\text{O}}_{2}$$

**FIGURE 4:**
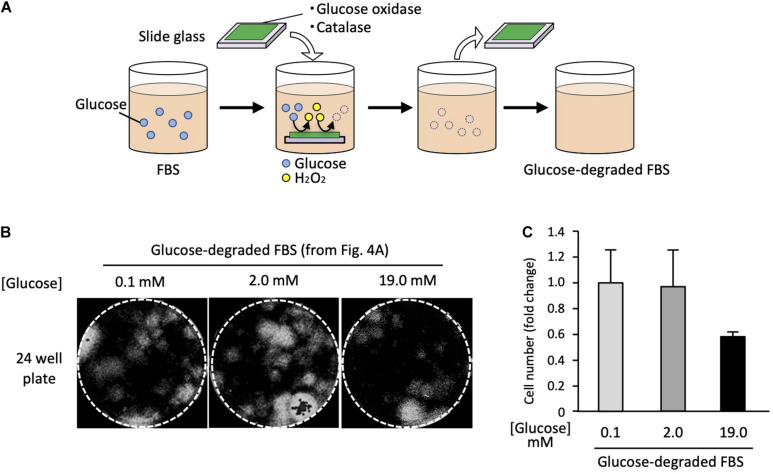
Satellite cells are able to grow in glucose-depleted medium. **(A)** Scheme of the enzymatic glucose degradation in FBS. To deplete the glucose included in FBS, GOD and catalase were immobilized on a glass slide and incubated with FBS. After incubation, enzymes immobilized slide glasses were removed. **(B)** Analysis of cell proliferation at different glucose concentrations. EDL derived from satellite cells were cultured in each different growth medium for 6 days, stained with DAPI, and analyzed by microscopy. **(C)** Cell numbers (derivation shown in Figure 4B) at different glucose concentrations. Values are presented as mean ± SEM (*n* = 2–4).

To remove the enzymes after the reactions, we immobilized them on a glass slide using a UV curable polymer. FBS was incubated with the enzymes immobilized on the glass slide (Figure 4A). After enzyme treatment, the final glucose concentration in the medium was 0.1 mM, which was less than 1% of that in the standard high-glucose medium.

Using the glucose-depleted medium (termed as very-low-glucose medium), we prepared three different media containing the following glucose concentrations: 0.1, 2.0, and 19.0 mM by adding glucose powder. Satellite cells from 20 myofibers were cultured in each medium for 6 days using 24-well plates. The cell numbers were evaluated by microscopy. Surprisingly, the satellite cells proliferated normally in the very-low-glucose medium (Figure 4B), and the cell number in this medium was similar to that in the low-glucose medium (2.0 mM) (Figure 4C). We also prepared a serum-free medium containing chemically defined supplements instead of FBS and tried to use it to culture satellite cells. However, the cells did not survive in this medium, suggesting that serum factors are essential for the proliferation of satellite cells (data not shown).

### AMPK Is Not Involved in Low Glucose-Promoted Cell Proliferation

**FIGURE 5:**
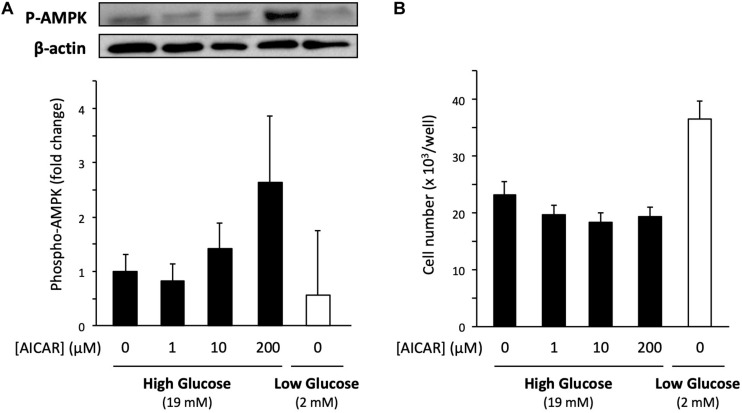
Effect of AMPK on the proliferation of primary satellite cells in low- and high-glucose media. **(A)** Amount of phosphorylated AMPK (P-AMPK) activated by AICAR in primary satellite cells derived from EDL cultured in the high-glucose medium for 6 days. The amount of P-AMPK in cells cultured in the low-glucose medium is also shown. P-AMPK was normalized to the expression of β-actin. Values are presented as mean ± SEM (*n* = 3). **(B)** Cell numbers in low- and high-glucose media after 6 days of cultivation. The cell number increased in low-glucose medium without AICAR administration. Values are presented as the mean ± SEM (*n* = 4–5).

To explore the molecular mechanism of low glucose-promoted cell proliferation, we studied AMP-activated protein kinase (AMPK), a serine/threonine protein kinase that plays a role in several signaling pathways by sensing the intracellular energy state. Because low glucose potentially decreases energy level of the cells, AMPK is expected to be involved in acting as a mediator of energy state-induced cell proliferation regulation (Mounier et al., 2015). To examine whether activation of AMPK increases the proliferation of cells cultured in the high-glucose medium, we used the AMPK activator 5-aminoimidazole-4-carboxamide ribonucleotide (AICAR). Primary cells cultured in the high-glucose medium were co-treated with 200 μM AICAR for 6 days. An increase in AMPK activation was confirmed by immunoblotting of the phosphorylation of threonine-172 in the AMPKα subunit, the major site of phosphorylation and activation of AMPK (Figure 5A). However, the cell number was not affected by the increased Phospho-AMPK (P-AMPK) (Figure 5B). In accordance with the abovementioned results, the P-AMPK level did not increase in the low-glucose medium (Figure 5A), in which cell proliferation was accelerated (Figure 5B). Taken together, these findings indicate that AMPK does not contribute to the increased cell proliferation activity under low glucose conditions.

## Discussion

Since glucose is a major energy source for cell proliferation, many cell types, such as cancer cells, benefit from using a sufficient amount of glucose (Wolf et al., 1992; Han et al., 2011; Jones and Schulze, 2012). Our finding that glucose restriction increases the proliferation of cultured muscle cells revolutionizes the existing concepts. DMEM containing 5 mM glucose is often used as a basic medium for culturing muscle cells (Elkalaf et al., 2013). However, in this study, we found that glucose-free DMEM (0 mM) improved the proliferation of satellite cells. Furthermore, we prepared glucose-depleted FBS to lower the glucose concentration in the medium, leading to a barely detectable glucose concentration (0.1 mM) in the culture medium. Although experiments to knock out glucose transporters (Glut1/4) are necessary to conclude that glucose governs cell proliferation, satellite cells were able to proliferate normally even under very low glucose conditions. Since FBS itself was found to be essential for cell survival, amino acids, lipids, and lactate may be alternative energy sources for cells.

The glucose concentration in standard high-glucose growth media seems to be physiologically too high compared to the blood glucose level in humans. The normal blood sugar level in fasting non-diabetics is under 6.1 mM (110 mg/dL) (American Diabetes Association, 2003), whereas the glucose concentration in the present high-glucose medium was 19 mM (342 mg/dL) and equivalent to the blood sugar level in patients with severe diabetes. In accordance with the physiological glucose levels, our *in vitro* experiments suggested that satellite cell proliferation was inhibited at glucose concentrations above 8 mM under adherent culture conditions; however, this effect was not observed in suspension culture. Diabetes mellitus is recognized as a risk factor for age-related muscle atrophy (sarcopenia) (Buford et al., 2010). Recent study showed that high glucose induces muscle atrophy via transcription factor KLF15 which act on differentiated myofibers (Hirata et al., 2019). In addition, our finding that high glucose levels inhibited cell proliferation and self-renewal gave us the idea that accelerated muscle atrophy in patients with diabetes is caused by satellite cell dysfunction due to hyperglycemia. Since blood glucose increases with age, independent of the development of diabetes (Basu et al., 2003; Oh et al., 2016), glucose-induced satellite cell impairment possibly causes sarcopenia.

Metabolic disorders, such as obesity and type two diabetes, are associated with reduced AMPK activity in skeletal muscles (Guan et al., 2016). Therefore, AMPK has been focused on as a potential factor to control muscle regeneration and mass in people with metabolic disorders. Previous studies reported that the satellite cell-specific deletion of AMPK reduced the proliferation and myogenic capacity of satellite cells during muscle regeneration (Fu et al., 2016; Theret et al., 2017), leading us to hypothesize that AMPK regulates the proliferation activity of satellite cells in response to glucose concentration. However, AMPK is probably not involved in the mechanism underlying the promotion of cell proliferation under limited glucose conditions because the level of phosphorylation of AMPK did not change and the activation of AMPK by AICAR did not enhance cell proliferation (Figure 5).

Although glucose restriction increased the number of cells in every population expressed Pax7, MyoD, and myogenin, the relative percentage of reserve cells, which were identified as Pax7+/MyoD-cells, was higher in low glucose medium. This means that lowering glucose concentration enhanced self-renewal of cultured satellite cells and delayed the differentiation, leading the maintenance of cell proliferation capacity. We also observed that Pax7 expression level was higher under the low glucose condition compared to high glucose (Figure 2C). Because the protein amount quantified by western blotting was normalized by β-actin level, this result indicates that the protein expression per a cell was upregulated by glucose restriction. Pax7 expression levels was known to be quickly down-regulated during satellite cell activation (Machado et al., 2017). Enhancements of myogenic factor expressions imply that the specificities of the cultured satellite cells were increased by lowering glucose concentration. High glucose potentially alters the DNA methylation status of several important genes which could regulate the cell proliferation and stemness of neural progenitor cells (Kandilya et al., 2020). Also, the other report suggested that glucose is required for histone acetylation in proliferating satellite cells and the determination of myogenic differentiation potential (Yucel et al., 2019). At present it is not clear whether low glucose medium directly downregulates the histone acetylation level in the cultured cells, or whether other factors such as pyruvate dehydrogenase (PDH) regulates them.

Primary cultures of satellite cells have widely been used to study the myogenesis, metabolism, myokine secretion, and contractile capacity of muscle cells (Lecompte et al., 2017; Sanchez et al., 2018; Chen et al., 2019). However, a disadvantage of satellite cells is their low proliferation activity compared to that of other muscle cell lines, such as C2C12 and L6; therefore, it takes effort to obtain a sufficient number of satellite cells for experimental use. We found that a low glucose concentration promotes the proliferation of satellite cells and may therefore overcome the abovementioned problem. Furthermore, cells cultured in the low-glucose medium could be passaged and frozen, indicating that this medium is valuable to experimenters. In particular, we found that contaminated other cells that extrude satellite cells can be eliminated in low-glucose media to obtain a pure satellite cell population, even from the soleus, a difficulty type of muscle for isolating satellite cells.

In summary, we showed here, that satellite cell proliferation is promoted by glucose restriction under adherent culture conditions. This finding suggests that a supra-physiological concentration of glucose directly inhibits the regeneration of skeletal muscles and facilitates sarcopenia in diabetes mellitus. More research is required to further elucidate the mechanism by which glucose restriction regulates satellite cell proliferation; identification and investigation of molecules that act as glucose sensors may be useful for this purpose.

## Data Availability Statement

The raw data supporting the conclusions of this article will be made available by the authors, without undue reservation.

## Ethics Statement

The animal study was reviewed and approved by the Experimental Animal Care and Use Committee of Tokyo Metropolitan University.

## Author Contributions

YF, YK, MA, and YMi performed and analyzed the experiments. YF, YMa, and NF designed the experiments and wrote the manuscript. All authors contributed to the article and approved the submitted version.

## Conflict of Interest

The authors declare that the research was conducted in the absence of any commercial or financial relationships that could be construed as a potential conflict of interest.
