# Supplementary material for: Excess Glucose Impedes the Proliferation of Skeletal Muscle Satellite Cells Under Adherent Culture Conditions
Source: Front Cell Dev Biol. 2021 Mar 1;9:640399. doi: 10.3389/fcell.2021.640399 (PMC7957019; doi:10.3389/fcell.2021.640399)
Supplement: Supplementary file 1 [file Data_Sheet_1.pdf]

Supplemental Files

Excess glucose impedes the proliferation of skeletal muscle satellite cells under adherent culture conditions

Yasuro Furuichi[1], Yuki Kawabata[1], Miho Aoki[1], Yoshitaka Mita[1], Nobuharu L. Fujii[1, 2] and Yasuko Manabe[1, 2]

[1]*Department of Health Promotion Sciences, Graduate School of Human Health Sciences, Tokyo Metropolitan University, Tokyo, Japan,*

[2]*Corresponding Authors*

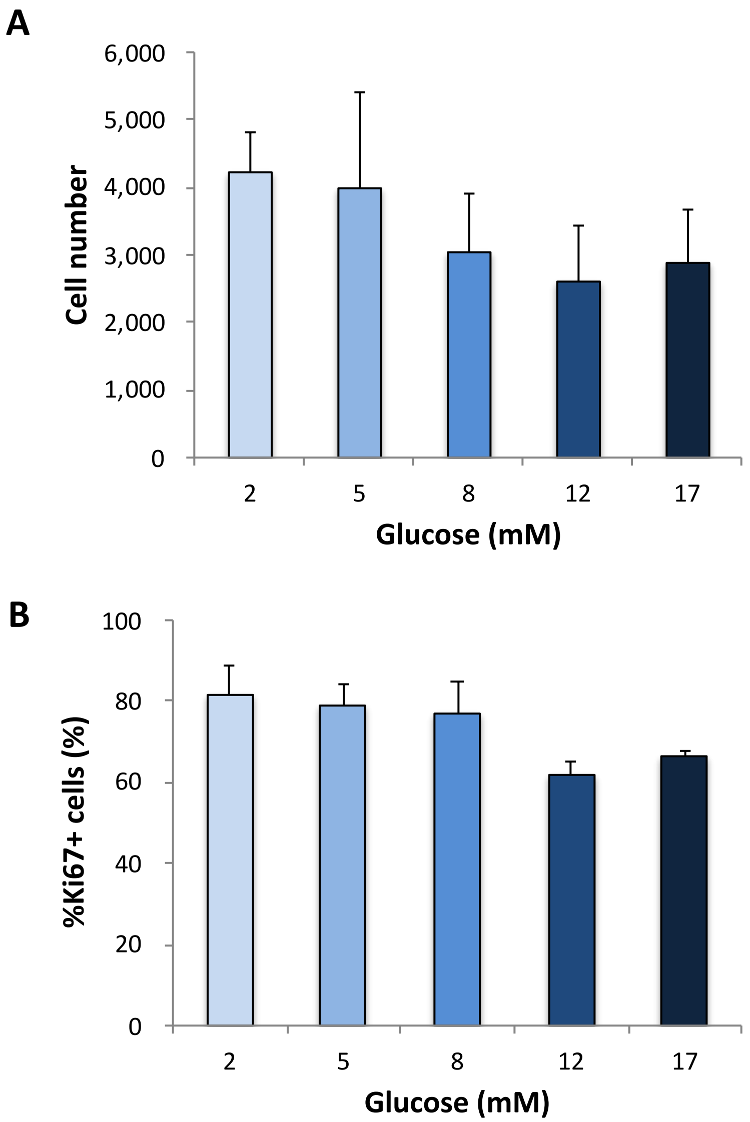

Supplemental Figure 1. Satellite cell proliferation under different glucose concentrations in medium.

Satellite cells derived from 20 myofibers of extensor digitorum longus (EDL) were grown in media with different glucose concentrations and stained with Ki67 antibody and DAPI. Cell numbers (A) and the percentage of Ki67-positive cells (B) were evaluated on day 6. Values are presented as mean ± SEM (n = 3). Although significant differences were not detected, cell proliferation was enhanced in media with a glucose concentration of 8 mM or less.

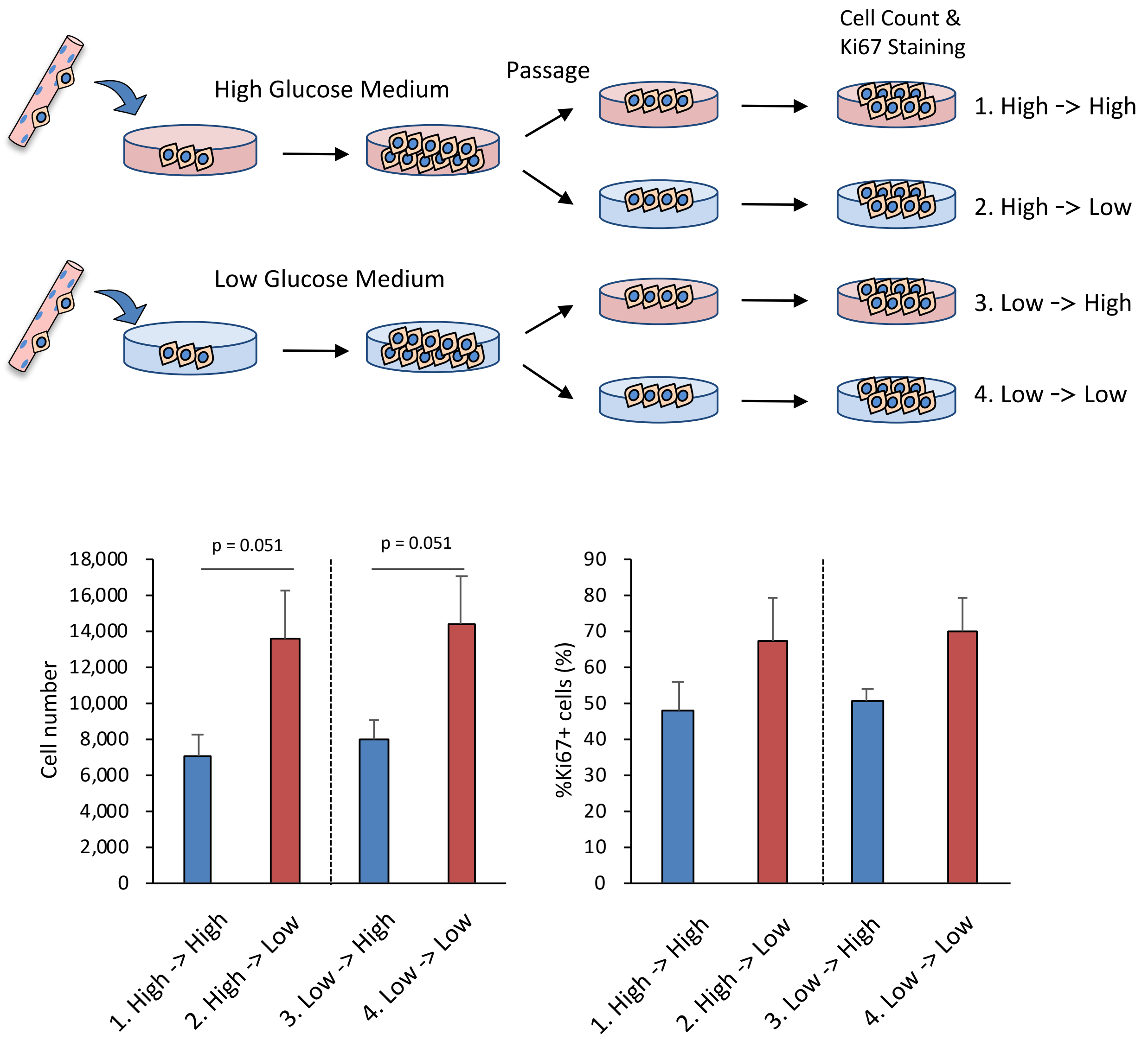

Supplemental Figure 2. Cell proliferation evaluation with equalized cell number. Satellite cells derived from the extensor digitorum longus (EDL) were grown in high-glucose or low-glucose media and passaged in 24-well plates at the same cell density. Then, the cells were cultured in high- and low-glucose media. “High -> High” indicates satellite cells were firstly cultured in the high glucose medium, then passaged to the high glucose medium. Similarly, High -> Low” indicates that satellite cells were first cultured in a high-glucose medium and then passaged to a low-glucose medium. After five days, the cells were stained with DAPI and Ki67 antibodies. Cell numbers and percentage of Ki67-expessing cells were counted. Cell numbers and percentage of Ki67+ cells tended to be higher in low-glucose medium than in high-glucose medium. Values are presented as mean ± SEM (n = 5).

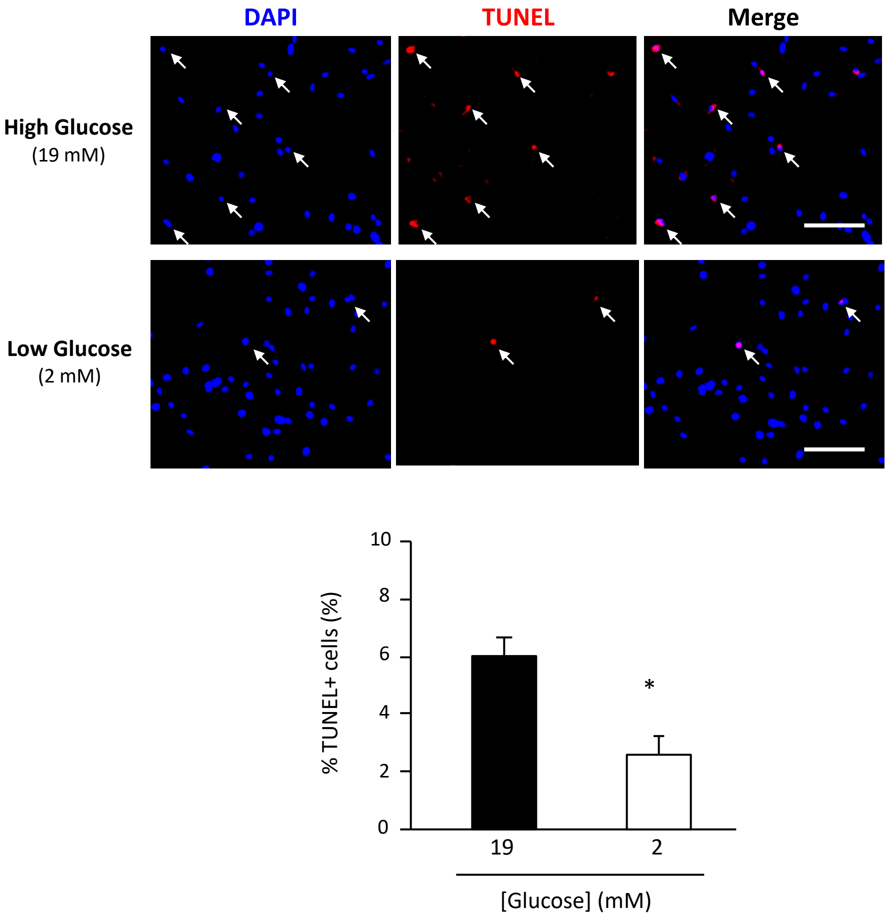

Supplemental Figure 3. Low-glucose growth medium decreases apoptosis of cultured satellite cells.

Satellite cells derived from the extensor digitorum longus (EDL) were grown in high-glucose or low-glucose media in 24 wells for 6-days. After fixation, the cells were stained with TUNEL (red) and DAPI (blue). TUNEL-positive cells were counted and normalized to all the cells. The proportion of TUNEL-positive apoptotic cells was lower in the cells cultured in the low-glucose medium than in the high-glucose medium. Scale bars are 100 μm. Values are presented as mean ± SEM (n = 4). *$p < 0.05$.

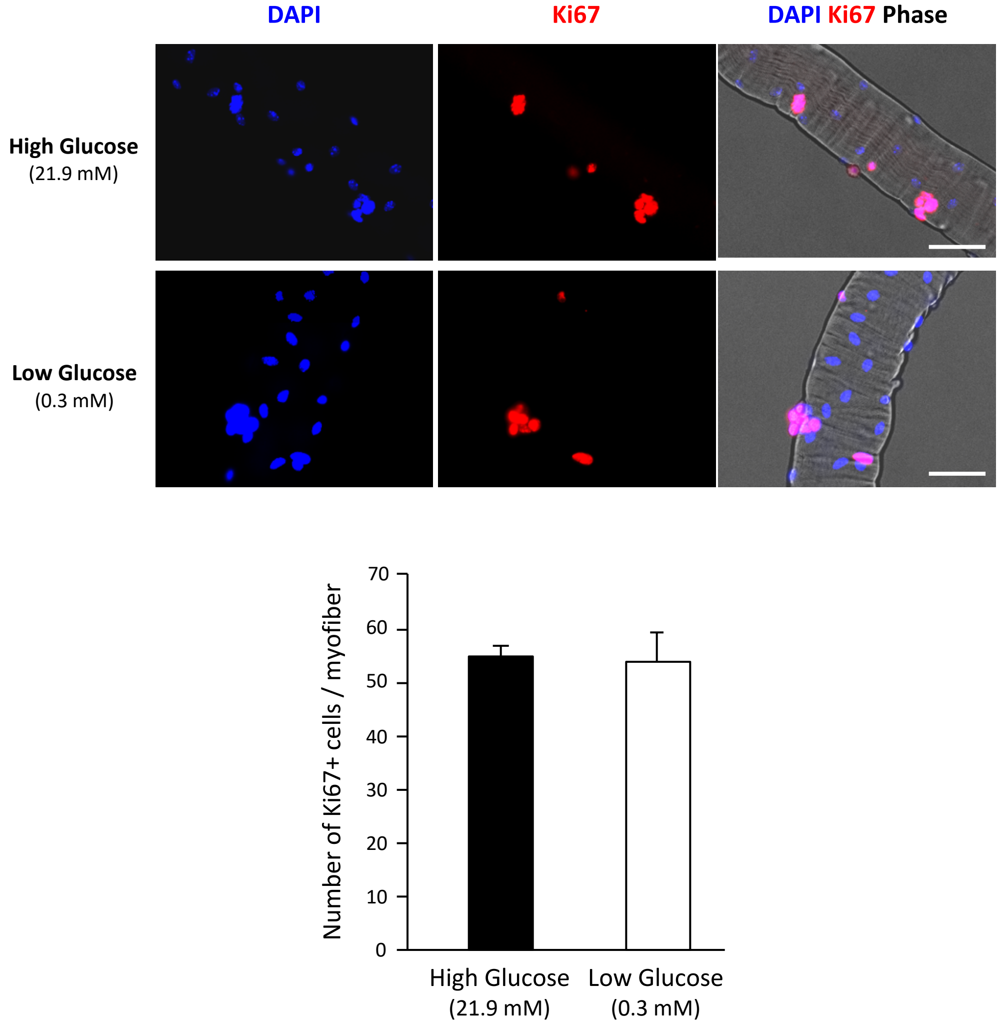

Supplemental Figure 4. Cell proliferation evaluation in fiber suspension culture. Isolated myofibers derived from extensor digitorum longus (EDL) were cultured in high-and low-glucose medium for 72 hours, and then satellite cells were stained for Ki67 (red) and DAPI (blue). The number of Ki67-positive cells per myofiber was counted and compared between high- and low-glucose media. There was no significant difference between glucose concentrations. Scale bars are 50 μm. Values are presented as mean ± SEM (n = 3).

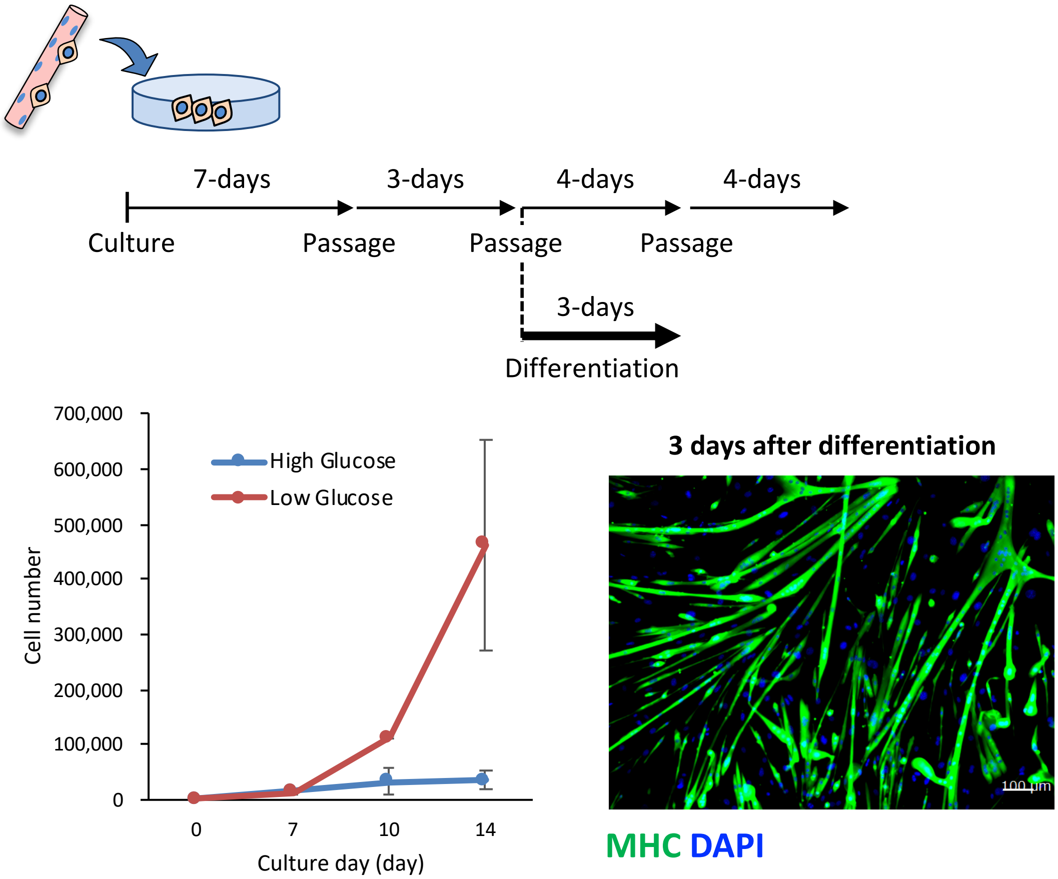

Supplemental Figure 5. The cells cultured in low-glucose medium can proliferate and differentiate after passages.
Satellite cells derived from the extensor digitorum longus (EDL) were grown in high-glucose or low-glucose media and passaged several times. Cell numbers were counted following the passage. While cell proliferation was inhibited after 7 days in high-glucose medium, the cells in the low-glucose medium maintained their proliferative abilities. After the cells were passaged twice, they were differentiated and stained with DAPI and myosin heavy chain (MHC), a marker of differentiation. The cells formed myotubes and expressed MHC normally.

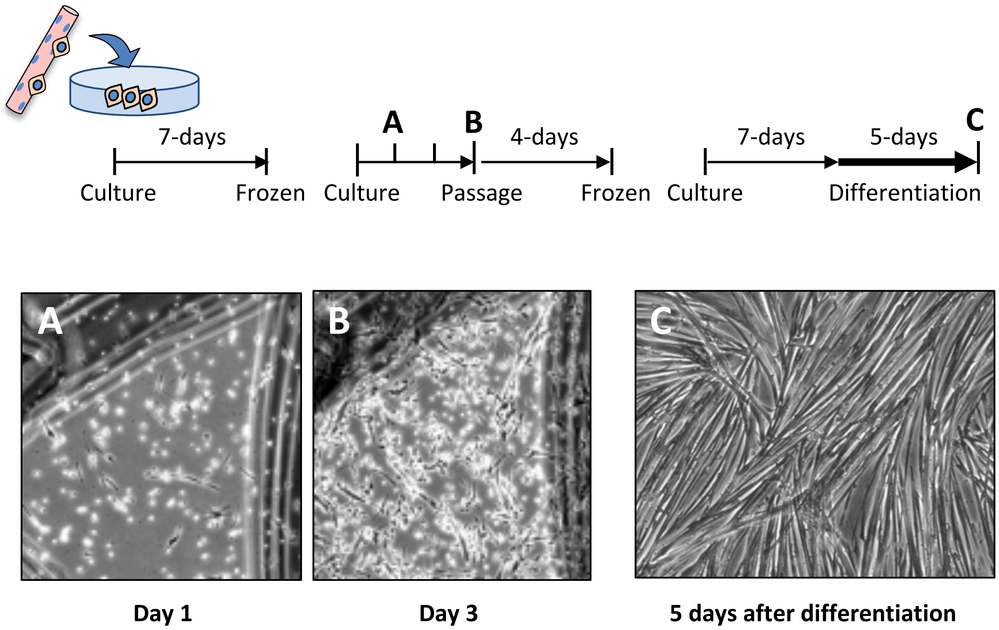

Supplemental Figure 6. Cells cultured in low-glucose medium can be frozen

Satellite cells derived from the extensor digitorum longus (EDL) were grown in low-glucose medium and frozen in cell storage solution (CELLBANKER2, BAMBANKER) following the manufacturer's instructions. The cells were thawed and cultured. The cells were photographed at the same point before (A) and after (B) 48 hours, indicating that the cells proliferated. The cells were able to differentiate normally after being frozen twice (C).

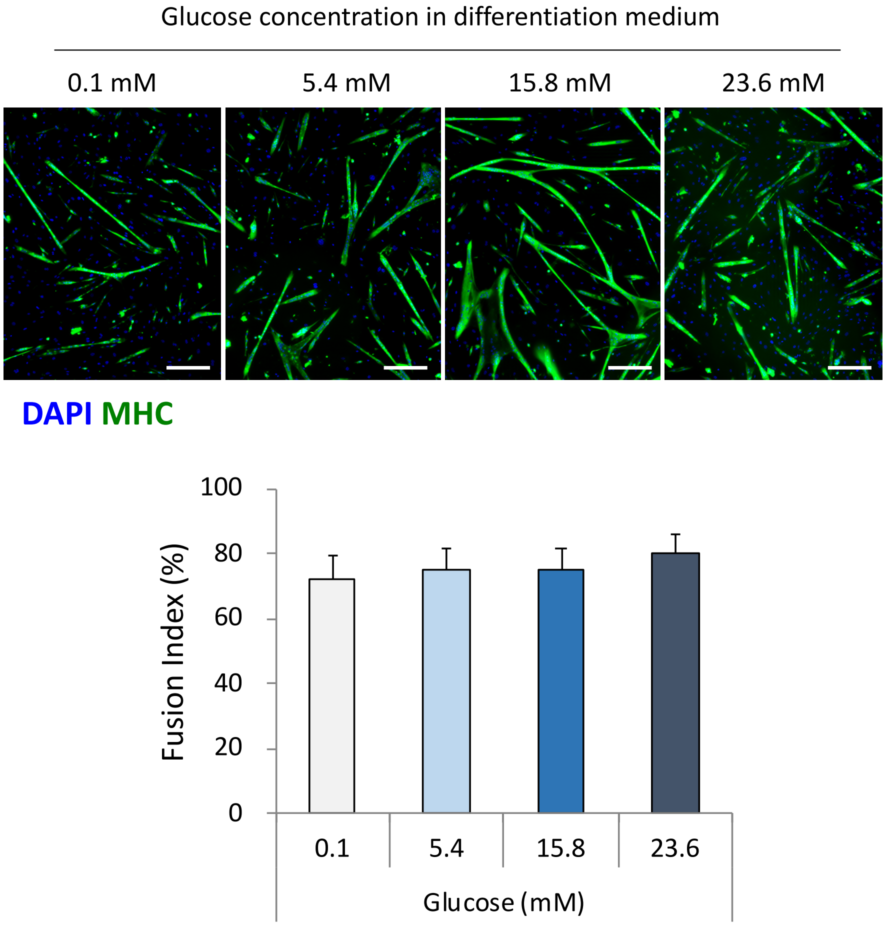

Supplemental Figure 7. Effect of glucose concentration on myoblast differentiation. Satellite cells derived from the extensor digitorum longus (EDL) were grown in low-glucose media and passaged in 24-well plates at the same cell density. Then, the cells were differentiated in 5% horse serum media (differentiation medium) with different glucose concentrations. The cells were stained for total myosin heavy chain (MHC) (green) and DAPI (blue). The fusion index, the percentage of nuclei inside myotubes of the total number of nuclei, was calculated and compared between glucose concentrations. There were significant differences among the different glucose concentrations. Values are presented as mean ± SEM (n = 4).

Table S1

Growth Medium Component

Name	unit	High Glucose Medium	Low Glucose Medium	Manufacturer	Product no.
FBS	%	30	30	BioWest	S1820
Antibiotic-Antimycotic	%	1	1	ThermoFisher	15240062
Chick Embryo Extract	%	1	1	USBiological	C3999
GlutaMAX™ Supplement	%	0	1	ThermoFisher	35050061
DMEM, high glucose, GlutaMAX™ Supplement, pyruvate	%	68	0	ThermoFisher	10569
DMEM, no glucose, no phenol red, no glutamine	%	0	67	ThermoFisher	A14430

Measurements of Growth Medium

	unit	High Glucose Medium	Low Glucose Medium
Final Glucose concentration	mM	19	2
Osmolarity	mOsmol/kg	329	318

Components of DMEM

Component	unit	DMEM for High Glucose Medium	DMEM for Low Glucose Medium
**Amino Acids**			
Glycine	mM	0.40	0.40
L-Alanyl-Glutamine	mM	3.97	0.00
L-Arginine hydrochloride	mM	0.40	0.40
L-Cystine 2HCl	mM	0.20	0.20
L-Histidine hydrochloride-H2O	mM	0.20	0.20
L-Isoleucine	mM	0.80	0.80
L-Leucine	mM	0.80	0.80
L-Lysine hydrochloride	mM	0.80	0.80
L-Methionine	mM	0.20	0.20
L-Phenylalanine	mM	0.40	0.40
L-Serine	mM	0.40	0.40
L-Threonine	mM	0.80	0.80
L-Tryptophan	mM	0.08	0.08
L-Tyrosine disodium salt dihydrate	mM	0.40	0.40
L-Valine	mM	0.80	0.80
**Vitamins**			
Choline chloride	mM	0.03	0.03
D-Calcium pantothenate	mM	0.01	0.01
Folic Acid	mM	0.01	0.01
Niacinamide	mM	0.03	0.03
Pyridoxine hydrochloride	mM	0.02	0.02
Riboflavin	mM	0.00	0.00
Thiamine hydrochloride	mM	0.01	0.01
i-Inositol	mM	0.04	0.04
**Inorganic Salts**			
Calcium Chloride (CaCl2) (anhyd.)	mM	1.80	1.80
Ferric Nitrate (Fe(NO3)3"9H2O)	mM	0.00	0.00
Magnesium Sulfate (MgSO4) (anhyd.)	mM	0.81	0.81
Potassium Chloride (KCl)	mM	5.33	5.33
Sodium Bicarbonate (NaHCO3)	mM	44.05	44.05
Sodium Chloride (NaCl)	mM	110.34	110.34
Sodium Phosphate monobasic (NaH2PO4-H2O)	mM	0.91	0.91
**Other Components**			
D-Glucose (Dextrose)	mM	25.00	0.00
Phenol Red	mM	0.04	0.00
Sodium Pyruvate	mM	1.00	0.00

Table S2

Glucose Concentration of Serum solutions

Name	Glucose Concentration		Manufacturer	Product no.	Lot no.
FBS (Fetal Bovine Serum)	6.66	mM	BioWest	S1820	S08993S1820
FBS (Fetal Bovine Serum)	5.35	mM	Nichirei	175012	19F00C
HS (Horse Serum)	2.63	mM	ThermoFisher	16050-130	1517704
CEE (Chick Embryonic Extract)	2.62	mM	USBiological	C3999	L19101658
